# Longitudinal Relationship Between Activities of Daily Living and Depression in Older Adults Based on Parallel Process Latent Growth Curve Model with Mediation

**DOI:** 10.3390/healthcare13040415

**Published:** 2025-02-14

**Authors:** Desheng Yan, Guangming Li

**Affiliations:** 1 Inner Mongolia Minzu Preschool Education College, Ordos 017000, China; 2 Key Laboratory of Brain, Cognition and Education Sciences, South China Normal University, Ministry of Education, Guangzhou 510631, China; 3 School of Psychology, Center for Studies of Psychological Application, Guangdong Key Laboratory of Mental Health and Cognitive Science, South China Normal University, Guangzhou 510631, China

**Keywords:** older adults, activities of daily living (ADL), depression, parallel process latent growth curve model (PP-LGCM), mediation effect

## Abstract

**Objective:** The parallel process latent growth curve model (PP-LGCM) was used to examine the longitudinal relationship between activities of daily living (ADL) and depression and further tested whether chronic diseases (CDs) were associated with depression via mediating variable ADL. **Methods:** A sample of 2014 Chinese older adults aged 60 and over from the China Health and Retirement Longitudinal Survey (CHARLS) was used. The activities of daily living scale, self-rating depression scale, and chronic diseases scale were used to investigate the ADL, depression, and CD levels of older adults. Following certain statistical analysis steps, we used SPSS 26 and Mplus 8.0 to perform statistical analysis on the data. **Results:** Firstly, ADL significantly declined in older adults from 2011 to 2018, while depression had a significant rise. Secondly, the intercept of ADL was correlated with the intercept of depression (*r* = 0.487, *p* < 0.001), and the slope of ADL was positively correlated with the slope of depression (*r* = 0.844, *p* < 0.001). Finally, the intercept of ADL mediated 39% of the association of chronic diseases and the intercept of depression. **Conclusions:** Our findings showed the trajectories of ADL and depression in older adults and demonstrated that ADL have various associations with depression in longitudinal development. In addition, the effect of chronic diseases on depression is partially mediated by ADL. The ADL play a partial mediating role between chronic diseases and depression in older adults, with an indirect effect of 39%, indicating that ADL are very important. Grasping the mediating mechanism of ADL will help alleviate depression levels in older adults with chronic diseases.

## 1. Introduction

The global aging trend is increasingly serious. According to the population estimation of the United Nations (UN), the proportion of people aged 65 and above in the global population may rise from 6.8% in 2000 to 14.3% in 2040, entering a stage of moderate aging. By 2050, it may rise to 16.3%, and by the middle to late stage for the second half of this century, it may reach 21%, entering a stage of severe aging. Across the globe, Asia will become one of the regions with the largest elderly population [1]. China will also enter a deeply aged society at an irreversibly high speed. Based on the data of the Chinese Seventh National Population Census, the proportion of the population aged 60 and above has reached 18.70%, up 5.44 percentage points, and the growth rate is significantly higher than that of 10 years ago, exceeding the proportion of children for the first time [2,3]. On 17 January 2025, the National Bureau of Statistics released the following data: according to preliminary accounting, in 2025, there will be 31.031 million people aged 60 and above, accounting for 22.0% of the national population, including 22.023 million people aged 65 and above, accounting for 15.6% of the national population [4]. In this aging background, various health problems of older adults, such as impaired activities of daily living (ADL), depression, and so on, have gradually attracted widespread attention from all walks of life.

With the increase in age, the physical function of older adults is increasingly impaired, and the impaired rate of ADL of older adults aged 85 years old and over in the United States is 20.7% [5,6], while that of Chinese older adults aged 60 years old and over is 32.2% [7]. Long-term impairment of ADL will not only affect the living of older adults but also make them feel increasingly incompetent, which may cause cognitive decline and decreased subjective well-being [8]. In addition to being vulnerable to reduced physical health, older adults are also prone to psychological problems. For older adults, keeping a high level of muscle strength in late life may be helpful to achieve successful aging [9]. Older adults participating in one or more leisure activities have greater odds for successful aging compared to those who did not participate in leisure activities [10]. Depression is a type of emotional psychological disorder characterized by low mood, which has become an important public health problem in recent years, with approximately 7% of the world’s older adults suffering from depression [11,12,13]. Depression can significantly reduce quality of life of older adults and increase their risk of suicide and death [14,15,16]. Therefore, ADL and depression are two health problems worth paying attention to in older adults. Moreover, ADL and depression belong to physical health and psychological health, respectively, and they cannot only act alone, but also affect each other.

### 1.1. The Cross-Sectional Studies of Relationship Between ADL and Depression

Many scholars in various countries have carried out cross-sectional studies on the relationship between ADL and depression in older adults [17]. A British study using data from the National Psychiatric Incidence Survey found that impaired ADL could increase the risk of depression, and there was a cumulative effect, that is, the more dimensions of impaired ADL, the greater the risk of depression [18]. Kim and Choi (2015) [19] investigated 210 Korean American older adults’ ADL and depression status and used a multilevel regression model to explore their relationship, reporting that better ADL could significantly predict a lower depression level. A study in the Netherlands also found similar results, in particular for self-care, where social participation was more associated with depression [20]. Ai et al. (2020) [21] conducted a cross-sectional study of 628 older adults from 13 communities in Wuhan, China, and found that there was a significant positive correlation between ADL score and depression score, and they shared common risk factors (e.g., educational background and hobbies). There are also clinical studies. For example, Na et al. (2017) [22] found that ADL in male older adults’ patients with first-episode depression were significantly reduced and improved after treatment. These cross-sectional studies showed significant association between ADL and depression in older adults.

### 1.2. The Longitudinal Studies of Relationship Between ADL and Depression

Compared with cross-sectional studies, there are few longitudinal studies about the relationship between ADL and depression. A study of 1116 Australian older adults, using ADL as a time varying covariate, found ADL were a very significant predictor of depression in older adults [23]. Another study based on data from the Amsterdam longitudinal study on aging found that, after controlling for demographic information and geriatric health status, older adults with chronic depression had a more significant decline in physical function than those without depression during a 3-year follow-up (OR [95%CI] = 2.83) [24]. He et al. (2019) [25] found that the poorer the initial level of ADK of Chinese middle-aged and older adults, the higher the depression level of themselves and their partners in the future. Although these longitudinal studies basically confirmed the significant correlation between ADL and depression in older adults, they all took one of the variables (ADL or Depression) as the main variable and the other as a covariate, so that such an approach could not further explore their relationship in longitudinal development.

Chen et al. (2012) [26] applied an autoregressive latent trajectory model (ALT) and parallel process latent growth curve model (PP-LGCM) to investigate the longitudinal relationship between ADL and depression in 442 older adults in the Taiwan community. At first, the study found that the initial level of ADL predicted the initial level of depression significantly, and vice versa. Secondly, the increased level of ADL significantly predicted the increased level of depression. But it should be noted that the sample data of Chen et al. are small and limited to urban older adults in Taiwan, so the inferences of the research results are limited.

### 1.3. ADL as a Mediator

Many studies have found that ADL are significantly associated with chronic diseases and depression, respectively [27,28,29,30]. Moreover, some scholars simultaneously studied the relationship among chronic disease, ADL and depression, and found that the ADL were the mediating factor in the process of chronic disease affecting depression. Fu (2018) [31] investigated 1318 older adults aged 60 and above in Beijing, and found that chronic diseases had a positive predictive effect on depression in older adults and they were partially mediated by ADL, with a mediating effect of 14%. Similarly, Xiao et al. (2021) [32] also found that chronic diseases had a significant impact on the mental health of older adults (e.g., anxiety and depression), and ADL played a partial mediating role in this process, with a mediating effect of 32.52%. Jiang et al. (2020) [33] found that middle-aged and older adults’ patients with chronic diseases or comorbidities are more prone to depressive symptoms, and ADL play different mediating roles in the influence of different chronic diseases on depression, among which ADL have the largest mediating role in the process of stroke affecting depression, with a mediating effect of 34.6%. Moreover, the mediating effect of ADL was 19.61% in the process of comorbidities on depression. However, these studies were all cross-sectional studies, lacking longitudinal studies.

### 1.4. The Present Study

To sum up, most of the previous studies on the relationship between ADL and depression in older adults are cross-sectional studies and are thus unable to explore their relationship in depth, although there are a few longitudinal studies with the limitations of statistical method and samples. As for the studies on the mediating relationship among chronic diseases, ADL and depression, there are all cross-sectional studies, lacking longitudinal ones. Therefore, the present study will use the data of the China Health and Retirement Longitudinal Survey (CHARLS) and relevant longitudinal statistical models (e.g., PP-LGCM with mediation) to explore the development trajectory of ADL and depression in older adults and their relationship in longitudinal development, and further explore the mediating effect of ADL between chronic diseases and depression.

## 2. Method

### 2.1. Participants

Participants were drawn from the CHARLS, which is a large-scale interdisciplinary survey project led by the National School of Development at Peking University, with the aim of collecting high-quality micro-data on families and individuals of middle-aged and older adults aged 45 and over for the purpose of analyzing population aging in China. It is an investigation and research recognized by the Ethics Committee of Peking University. The researchers behind the investigation of the CHARLS obtained the consent of all participants and there are no moral issues. Our study was approved by the South China Normal University (SCNU) research ethics board (Institutional Review Board) who approved the experiments, including any relevant details, and confirmed that all experiments were performed in accordance with relevant guidelines and regulations.

The national baseline survey of the CHARLS was carried out in 2011 and tracked almost every two years. So far, there have been four survey waves (2011, 2013, 2015 and 2018) in 150 counties of 28 provinces (autonomous regions and municipalities directly under the Central Government). The survey was carried out in 450 communities (villages), covering about 17,000 middle-aged and older adults across the country. The survey contents included personal basic information, family structure, health status and physical measurement, and so on, which had good representativeness [34].

There were 7669 older adults aged 60 and over who participated in the CHARLS in 2011. In this study, older adults aged 60 and over who participated in four waves were selected, and samples with lack of follow-up and death or failure to response the items were deleted. The following three types of data were not retained: (1) values exceeding three standard deviations, such as some participants reaching the age of 999; (2) obvious logical errors, such as some genders being both male and female; (3) suspicious data, such as clearly and systematically filled in responses, i.e., 1234, 1234, 1234, etc. At last, a total of 2014 effective participants were obtained. For older adults in 2014, their average age was approximately 67.6 years, with a standard deviation of 6.5 years. Among them, there were 1004 males, accounting for 49.9%, and 1010 females, accounting for 50.1%; there were 957 older adults in urban areas, accounting for 47.5%, and 1057 older adults in rural areas, accounting for 52.5%. The characteristics of the study participants among older adults in 2014 are shown in Table 1.

### 2.2. Measurements

#### 2.2.1. Activities of Daily Living Scale

The activities of daily living scale was used to evaluate ADL of older adults [35]. The measurement indicators included dressing, bathing, eating, transfer, toilet and continence. All items have four responses (1 = no difficulty, 2 = difficulty but still able to complete, 3 = difficulty and need help, 4 = unable to complete). The total score of these six items was taken as the total score of ADL of older adults, which ranged from 6 and 24 points, with a higher score indicating worse ADL. The Cronbach’s alpha ranged from 0.71 to 0.76 across the four waves.

#### 2.2.2. Self-Rating Depression Scale

The Center for Epidemiologic Studies Depression Scale (CES-D10) was used to reflect the depression status of older adults, which has good reliability and validity in the older adult population in China [36]. The CES-D10 mainly surveys the psychological feelings and behaviors of older adults in the past week, which has 10 items in total, including 8 negative indicators (e.g., “I am bothered by some small things”) and 2 positive indicators (e.g., “I am very happy”). All items have four responses (“Little or none”, “not too much”, “sometimes or half the time”, “Most of the time”). Consistent with the scoring method of previous studies [37], the score of every item ranged from 0 to 3 in this study, with the total sore ranging from 0 to 30. With the reverse scoring of two reverse scoring items, a higher total score of depression indicated more severe depression status. The Cronbach’s alpha ranged from 0.75 to 0.80 across the four waves.

#### 2.2.3. Chronic Diseases Scale

Chronic diseases were identified according to self-reported physician diagnosis. The measurement indicators included 14 chronic diseases: hypertension, hyperlipidemia, diabetes, cancer or malignant tumor, chronic lung disease (e.g., chronic bronchitis, emphysema), liver disease, heart disease (e.g., coronary heart disease, angina, congestive heart failure), stroke, kidney disease, stomach or other digestive disease, emotional or psychiatric problems, memory-related disease, arthritis or rheumatism, and asthma. The number of chronic diseases was calculated according to the self-report provided by participants. Total sore ranged from 0 to 14, with a higher sore indicating more chronic diseases. This study adopted the data of chronic diseases measured at wave 1. The final comorbidities presented were the prevalent ones indicated by the participants or answered based on questions and they were self-reported.

### 2.3. Analysis

SPSS 26.0 was used to conduct basic statistical analysis. Three latent growth curve models (LGCMs) were used to examine the research questions, utilizing Mplus 8.0 [38]. These analysis steps were as follows:

Step 1: Descriptive statistics analysis. This section mainly includes the analysis of the mean, standard deviation, and correlation coefficient for each stage. The main purpose is to analyze basic information of some variables and deduce whether there are significantly positive or negative correlation among ADL, depression and chronic diseases for all waves or not.

Step 2: LGCM analysis. A univariate LGCM was used to test the developmental trajectories of ADL or depression. The univariate LGCM had two latent growth factors: (1) intercept, a constant for any individual across time, representing the initial level of developmental trajectory of ADL or depression at Time 1; (2) slope, representing the rate of change developmental trajectory of ADL or depression over time. The intercept and slope factors all have a mean and variance that can be estimated from the data [39]. Significant results of the mean suggest that there are intra-individual changes, while significant results of the variance suggest that inter-individuals are quite different at the initial level and the rate of change across the whole samples [40]. The loading of the intercept factor at each time point was fixed as 1, while that of the slope factor was fixed as 0, 1, 2, and 3, which represents linear growth.

Step 3: PP-LGCM analysis. A parallel process latent growth curve model was used to test the longitudinal relationship of ADL and depression through the combination of two univariate LGCMs. This study examined the association between (1) the initial levels of ADL and the initial levels of depression; (2) the change rate of ADL and the change rate of depression; (3) the initial levels of ADL and the change rate of depression; (4) the initial levels of depression and the change rate of ADL.

Step 4: PP-LGCM analysis with mediation. A PP-LGCM with mediation was executed to test the mediating relationship among chronic diseases, ADL and depression. In this study, chronic disease was the independent variable, and depression was the outcome variable, while ADL was a mediator. The mediating process in PP-LGCM was defined as the independent variable (i.e., chronic disease) influencing the growth of the mediator (i.e., ADL), which, in turn, affected the growth of the outcome (i.e., depression) [41].

For chronic disease, ADL and depression, the Kolmogorov–Smirnov (K-S) test found that all data were normally distributed (Z = 0.781, *p* = 0.681; Z = 0.701, *p* = 0.601; Z = 0.612, *p* = 0.581) [42]. The maximum likelihood robust estimation was used to deal with the data. In this study, model fit was evaluated based on the comparative fit index (CFI), the Tucker–Lewis index (TLI), the mean square error of approximation (RMSEA), and standardized root-mean-squared residual error (SRMR). As suggested by prior researchers, model fit is considered to be good when χ^2^/*df* ≤ 5; CFI ≥ 0.90, TLI ≥ 0.90, RMSEA ≤ 0.08, and SRMR ≤ 0.08 [43,44].

## 3. Results

### 3.1. Descriptive Statistics

Table 2 showed the descriptive statistics for the samples across four waves.

From Table 2, it can be observed that the mean ranged from 6.79 to 7.28 for ADL, seeming to increase gradually from wave 1 to wave 4. For depression, the mean ranged from 9.73 to 11.03, seeming to increase gradually from wave 2 to wave 4. And for chronic diseases at wave 1, the mean is 1.98 with a standard deviation of 1.56. For all waves, there are significantly positive correlations among ADL, depression and chronic diseases.

### 3.2. Estimates for LGCMs

Estimates for univariate LGCMs are shown in Table 3. The univariate LGCM estimates demonstrated good fit to the data (χ^2^ (5) = 12.26, χ^2^/*df* = 2.45, CFI = 0.98, TLI = 0.98, RMSEA = 0.03, and SRMR = 0.03 for ADL; χ^2^ (5) = 13.06, χ^2^/*df* = 2.61, CFI = 0.95, TLI = 0.94, RMSEA = 0.05, and SRMR = 0.04 for depression).

As shown in Table 3, the mean intercept and slope of ADL and depression in older adults were statistically significant, indicating that both of them showed significant linear growth across waves. For ADL, the mean value of intercept was 6.732 (*t* = 198.777, *p* < 0.001), suggesting that the initial level of ADL was 6.732. And the mean value of the slope was 0.175 (*t* = 11.402, *p* < 0.001), which can be interpreted as the average increase in ADL per unit of time. The variance of the intercept and slope for ADL was 1.225 (*t* = 5.398, *p* < 0.001) and 0.111 (*t* = 2.506, *p* < 0.05), respectively, suggesting there was significant variation in the initial levels and the rate of change across individuals. In terms of depression, the mean value of intercept was 10.190 (*t* = 74.663, *p* < 0.001), which represented the baseline of depression. And the mean value of the slope was 0.170 (*t* = 3.282, *p* < 0.01), indicating that the score of depression increased with a mean speed of 0.170 per wave. The variance of the intercept and slope for depression was 20.618 (*t* = 16.340, *p* < 0.001) and 0.899 (*t* = 3.506, *p* < 0.001), respectively.

### 3.3. Estimates for PP-LGCM

**Figure 1:**
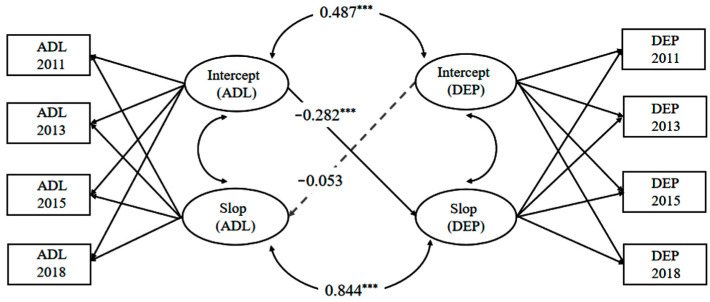
Parallel process latent growth curve model of the relationship between ADL and depression. Note: ADL = activities of daily living; DEP = depression; *** *p* < 0.001.

In order to explore the relationship between ADL and depression in the longitudinal development of older adults, we constructed a PP-LGCM, and the fitting results were acceptable (χ^2^ (24) = 109.51, χ^2^/*df* = 4.56, CFI = 0.95, TLI = 0.94, RMSEA = 0.06, and SRMR = 0.03). Estimates for linear LGCM with parallel process are shown in Table 4 and Figure 1.

As shown in Table 4 and Figure 1, there was a significantly positive correlation between the intercept of ADL and the intercept of depression (*r* = 0.487, *t* = 12.953, *p* < 0.001). There was also a significantly positive association between the slope of ADL and the slope of depression (*r* = 0.844, *t* = 6.135, *p* < 0.001), suggesting that the rapid impairment in ADL might also be accompanied by a rapid increase in depression. In addition, the intercept of ADL significantly predicted the slope of depression over time (β = −0.282, *t* = −4.440, *p <* 0.001), while the intercept of depression did not significantly predict the slope of ADL (β = −0.053, *t* = −0.743, *p* = 0.457).

### 3.4. Estimates for PP-LGCM with Mediation

**Figure 2:**
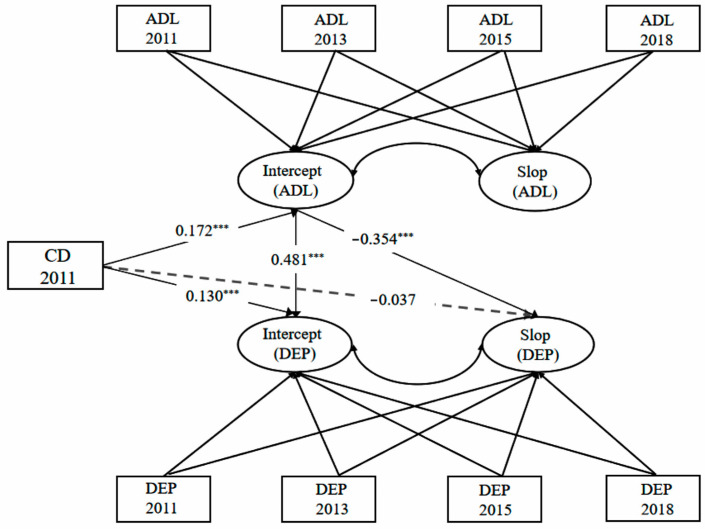
The mediating role of ADL. Note: ADL = activities of daily living; DEP = depression; CD = chronic diseases; *** *p* < 0.001.

A PP-LGCM with mediation was used to examine whether the effect of chronic diseases on the intercept and slope of depression was due to the intercept and slope of ADL. Chronic diseases are the independent variable, the ADL are the mediating variable and depression is the dependent variable in the PP-LGCM with mediation. The model estimates demonstrated an acceptable fit to the data (χ^2^ (27) = 119.12, χ^2^/*df* = 4.41, CFI = 0.95, TLI = 0.93, RMSEA = 0.05 SRMR = 0.03). The mediating role of ADL is shown in Figure 2.

As shown in Figure 2, chronic disease significantly predicted the intercept of ADL (β = 0.172, 95% CI [0.108, 0.236], *p* < 0.001), and the intercept of ADL significantly predicted the intercept of depression (β = 0.481, 95% CI [0.389, 0.572], *p* < 0.001). Meanwhile, chronic diseases significantly predict the intercept of depression (β = 0.130, 95% CI [0.065, 0.195], *p* < 0.001). A positive indirect association of chronic diseases via the intercept of ADL with the intercept of depression (indirect effect = 0.083, 95% CI [0.042, 0.134], *p* < 0.001) was found. After decomposing the effect proportion, the intercept of ADL mediated 39% of the association of chronic diseases and the intercept of depression was found (seeing Table 5).

As shown in Figure 2, the intercept of ADL significantly affects the slope of depression (β = −0.354, 95% CI [−0.408, 0.036], *p* < 0.001), indicating that the development of each stage is different, and the impact of ADL is not uniform, but gradually decreases, which may also be related to the accelerated functional decline in older adults. However, the impact of chronic diseases on depression is not significant (β = −0.037, 95% CI [−0.108, 0.137], *p* > 0.05), which may be related to the role of ADL.

## 4. Discussion

### 4.1. Longitudinal Relationship Between ADL and Depression in Older Adults

The current study focused on the trajectory of ADL and depression in older adults, and examined the longitudinal relationship between ADL and depression, while testing chronic diseases as a mediator of this association. Our longitudinal evidence suggested older adults showed a significant decline in ADL over time, although the initial level of ADL was not as low in 2011. Many cross-sectional studies have also shown that ADL are significantly associated with age, that is, ADL declined with aging [45,46]. As for depression, Chinese older adults showed a significant rise with a relatively high initial level, corresponding to many studies suggesting that age is a risk factor for depression in older adults.

Moreover, the current study found that ADL were significantly associated with depression in longitudinal development in older adults, using a parallel process latent growth curve model. Firstly, our findings showed that older adults who reported higher initial levels of ADL and reported higher initial levels of depression, which is in accordance with previous cross-sectional studies suggesting that older adults with impaired ADL were more prone to depression [47,48,49,50]. Secondly, accelerating impairment in ADL was associated with a rapid increase in depression over 8 years, implying that ADL and depression interact longitudinally in older adults. This result is consistent with the study of Chen et al. (2012) [26], which showed that changes in ADL could significantly predict changes in depression. Chen et al. (2012) [26] also found that initial levels of ADL did not significantly predict changes in depression, while initial levels of depression also did not significantly predict changes in ADL, which is in line with our study. However, the difference is that our findings showed that initial levels of ADL significantly negatively predicted changes in depression. This may be due to the fact that compared with the study of Chen et al. (2012) [26], the number and representativeness of data samples used in our study are better, which assists in finding a relationship between ADL and depression in older adults. But it is important to note that this result was actually different from what we expected, and we thought that it might be because worse initial levels of ADL were associated with higher initial levels of depression, and the latter might result in a slower subsequent increase due to the ceiling effect.

### 4.2. Longitudinal Relationship Between ADL and Depression in Older Adults with Mediation

According to the PP-LGCM with mediation, chronic diseases can be seen as the independent variable (X), ADL can be seen as the mediating variable (M) and depression can be seen as the dependent variable (Y). Chronic diseases (X) have a direct impact on depression (Y) and can be considered as a direct effect. However, if chronic diseases (X) have an effect on depression (Y) through the ADL (M), it can be considered an indirect effect.

In this paper, ADL play a partial mediating role between chronic diseases and depression in older adults using the PP-LGCM with mediation, with an indirect effect of 39%, indicating that ADL are very important. It shows that understanding the mediating mechanism of ADL will help alleviate depression levels in older adults with chronic diseases. In fact, some previous studies revealed that some chronic diseases could exert an indirect effect on initial levels of depression through initial levels of ADL in older adults [31,32,33]. Consistent with previous studies [27,28,51,52,53], our findings showed that the number of chronic diseases was positively correlated with ADL, meaning that the higher the number of chronic diseases, the worse ADL would be. The impairment of ADL affects basic daily activities of older adults and their participation in physical exercise and social activities, requiring carers over long-term periods. All these factors affect the self-confidence and self-esteem of older adults, reduce their sense of self-worth, and produce anxiety, depression and other negative emotions [54]. This suggests that when addressing older adults with chronic diseases, especially those with multiple chronic diseases (i.e., comorbidities), people need to pay attention not only to their impaired ADL, but also to the possible subsequent psychological problems. When caring for these older adults, depression can be prevented to a certain extent by helping them gradually alleviate or recover impaired ADL by increasing physical training [55].

### 4.3. Some Suggestions

This article is based on the “positive aging theory” [56] and the “positive activity theory” [57]. (1) The positive aging theory emphasizes viewing older adults and their lives from a positive perspective, respecting and inspiring their subjectivity and autonomy. Good functional ability means that older adults can maintain their independence in life, establish and maintain various relationships, continue to integrate into society, and realize personal value. (2) The positive activity theory emphasizes the active participation of older adults. Older adults with high levels of activity may be more likely to feel satisfied with life and adapt better to society than those with low levels of activity. It highlights that older adults can improve their emotional depression caused by the interruption of their social roles through new participation and roles, and re-recognize themselves in new social participation, thereby minimizing the distance between themselves and society.

Strengthening ADL in older adults can help improve two aspects of function: (1) slow down the deterioration of chronic diseases, and help improve one’s own immunity; (2) shift the attention of older adults, so that older adults do not focus on chronic diseases for a long time, which can lead to depression. Performing more daily activities can help older adults slow down the deterioration of mental illness such as depression. For this, it needs the joint efforts of the government, the family, the community, the society, and the older adults themselves to protect the physical and mental health of older adults.

Firstly, the government should play an important leading role in maintaining their ADL and enhancing their subjective well-being for older adults. The government should establish corresponding legal policies to protect the rights and interests of older adults. Relevant government departments should promote the development of the ADL of older adults effectively and improve their emotional state to enhance their quality of life in their later years. The government needs to strengthen the medical security of older adults, prevent them from developing various diseases, and to a certain extent ensure their physical health. Some specialized institutions to enhance the mental health of older adults can be established. For example, the government can provide professional psychological counseling services for older adults free of charge to help them solve their psychological problems such as depression.

Secondly, the family should provide corresponding ADL for older adults to enhance their physical function over the long term. Family members should provide full care and support for older adults. Families can hold regular activities to encourage the participation of older adults. This not only enriches the later life of older adults, but also allows them to obtain social support through activities in addition to family members such as spouses, and children. Older adults should also actively interact with those around them and establish good interpersonal relationships, which could help older adults to benefit from from the process of interacting with others and reduce the influence of negative emotions. Corresponding support provided by the family could help older adults maintain a happy mood and avoid developing depressive emotions.

Thirdly, the community should encourage older adults to persist in exercise and try to avoid putting older adults in a state of depression. The joint efforts of the community and individuals can be combined to arrange exercise for older adults. The community should offer guidance, popularize basic knowledge of physical exercise to the older adults, and encourage them to actively participate in sports activities. In addition, it is necessary to strengthen the construction of comprehensive sports and entertainment facilities, add suitable venues and equipment for older adults’ activities, and effectively create a good environment for older adults’ physical exercise to meet their fitness needs. The community can guide older adults to choose appropriate sports based on their own health level, such as jogging, Tai Chi, walking, etc., to cultivate interests and stimulate interest in life. More attention needs to be paid to the increase in physical fitness for preventing or improving the cognitive dysfunction of older adults in the community [58].

Fourthly, society should encourage older adults to continue learning and enable them to improve their ADL. Studying at universities for older adults can help them to keep up with the pace of the times, relieve negative emotions in their hearts, and develop the habit of using their brains frequently, reducing the risk of physical impairment. Therefore, society should vigorously promote the development of universities for older adults and improve the corresponding curriculum system construction. In addition, targeted training for the ADL of older adults can effectively enhance their immunity and alleviate chronic diseases. Older adults can receive training at home through devices such as mobile phones and computers. The more the training tasks meet the needs of daily life and the more physical functions are utilized, the more training effects can be transferred to daily life. In this way, depression in older adults can be alleviated.

Finally, older adults themselves should maintain a positive and optimistic attitude in the face of aging. Older adults should learn to accept the decline in their physical functions, adapt to changes in social roles, and take positive actions to adapt to a new life. When encountering difficulties in life, older adults should strive to adjust their mentality without depression, actively seek solutions, and seek help from others. In order to establish a correct attitude towards life, older adults should turn to psychological regulation and relaxation skills and enhance their confidence and self-efficacy. Only through their own continuous efforts of older adults can ADL be enhanced and depression levels can be reduced.

### 4.4. Innovation and Limitations

The strengths of our study include three aspects. First of all, the data from CHARLS, which had a representative large sample of the Chinese population aged 60 and over, were used. Secondly, a parallel process latent growth curve model was used to test the longitudinal relationship between ADL and depression in Chinese older adults. Thirdly, to our knowledge, this is the first study to examine the mediating relationship among chronic diseases, ADL and depression using a parallel process latent growth curve model with mediation. And we have concluded that the ADL play a partial mediating role between chronic diseases and depression in older adults.

However, some limitations also need to be mentioned. First, ADL in this study refer to basic activities of daily living (BADL), not including instrumental activities of daily living (IADL), so it is not clear whether the present result equally applies to the relationship between BADL and depression. Second, only the relationship among the number of chronic diseases, ADL and depression was explored, and the types of chronic diseases, which led to the inclusion of some chronic diseases not related to ADL and depression and may affect the results, were not distinguished. Further research can be conducted based on the above two points. Third, all data were self-reported and self-evaluated for older adults. Future studies should include objective measures to assess chronic diseases, ADL and depression. The results obtained may have certain biases. Last but not least, the patterns of trajectories for ADL and depression in Chinese older adults are heterogeneous, suggesting that there might be different typologies of the parallel process of ADL and depression [59].

## 5. Conclusions and Implications

(1) ADL in older adults are increasingly impaired with age, and the level of depression in older adults gradually increases with age. Using univariate latent growth curve models, our findings showed the trajectories of ADL and depression in older adults, respectively. With age, various functions of older adults show a declining trend, and ADL decrease. This may lead to worsening depression in older adults.

(2) Only a unidirectional relationship was found for the longitudinal relationship between ADL and depression in older adults based on PP-LGCM. The initial level of ADL is significantly correlated with the initial level of depression. There is a significant correlation between the level of growth in ADL and the level of growth in depression. The initial level of ADL can significantly predict the growth level of depression, but the initial level of depression can significantly predict the level of growth in ADL.

(3) The ADL in older adults play a partial mediating role in the process of chronic diseases affecting depression. There may not necessarily be a correlation between chronic diseases and depression in older adults, unless the chronic disease causes physical functional discomfort. However, chronic diseases related to impaired ADL in older adults, such as arthritis and stroke, are significantly associated with depression, indicating that the ADL in older adults may be a mediating factor in the impact of chronic diseases on depression.

In short, with the increasing age of older adults, their ADL decreases, but their chronic diseases and depression increase. However, through this study, we found that the true partial cause of depression in older adults is the ADL, rather than chronic diseases themselves. This suggests that people should pay attention to the enhancement of ADL in older adults. The government, family, community, society and older adults themselves should all take active action to improve their ADL and relieve their depression in older adults with chronic diseases.

## Figures and Tables

**Table 1:** Characteristics of the study participants among 2014 older adults.

Variables	Number of Participants	2014
Age	Age, *M* (*SD*)	*M* = 67.6 (*SD* = 6.5)
Gender	Male, *n* (%)	1004 (49.9%)
	Female, *n* (%)	1010 (50.1%)
Region	Urban areas, *n* (%)	957 (47.5%)
	Rural areas, *n* (%)	1057 (52.5%)

**Table 2:** Characteristics of the study participants among 2014 Chinese older adults across 4 waves.

Variables	1	2	3	4	5	6	7	8	9
1. ADL at wave 1	1.00								
2. ADL at wave 2	0.43 **	1.00							
3. ADL at wave 3	0.39 **	0.47 **	1.00						
4. ADL at wave 4	0.35 **	0.41 **	0.47 **	1.00					
5. DEP at wave 1	0.26 **	0.22 **	0.20 **	0.18 **	1.00				
6. DEP at wave 2	0.13 **	0.26 **	0.20 **	0.17 **	0.50 **	1.00			
7. DEP at wave 3	0.15 **	0.21 **	0.30 **	0.19 **	0.47 **	0.55 **	1.00		
8. DEP at wave 4	0.14 **	0.18 **	0.21 **	0.27 **	0.44 **	0.50 **	0.55 **	1.00	
9. CD at wave 1	0.13 **	0.09 **	0.11 **	0.11 **	0.17 **	0.11 **	0.12 **	0.11 **	1.00
*M*	6.79	6.81	7.12	7.28	10.80	9.73	10.44	11.03	1.98
*SD*	1.72	1.67	2.05	2.18	6.68	6.07	6.94	7.06	1.56

Note: ADL = activities of daily living; DEP = depression; CD = chronic diseases; ** *p* < 0.01.

**Table 3:** Estimates for univariate latent growth curve models (LGCMs).

		Estimate	*S.E.*	*t*	*p*
**ADL**	**Mean**				
	I_(ADL)_	6.732 ***	0.034	198.777	<0.001
	S_(ADL)_	0.175 ***	0.015	11.402	<0.001
	**Variance**				
	I_(ADL_)	1.225 ***	0.227	5.398	<0.001
	S_(ADL)_	0.111 *	0.044	2.506	0.012
**Depression**	**Mean**				
	I_(depression)_	10.190 ***	0.136	74.663	<0.001
	S_(depression)_	0.170 **	0.052	3.282	0.001
	**Variance**				
	I_(depression)_	20.618 ***	1.262	16.340	<0.001
	S_(depression)_	0.899 ***	0.256	3.506	<0.001

Note: I_(ADL)_ = the intercept of activities of daily living; I_(depression)_ = the intercept of depression; S_(ADL)_ = the slope of activities of daily living; S_(depression)_ = the slope of depression; * *p* < 0.05; ** *p* < 0.01; *** *p* < 0.001.

**Table 4:** Estimates for PP-LGCM.

		*CI* (95%)				
	β	Lower	Upper	*S.E.*	*t*	*p*
I_(ADL)_ ↔ I_(depression)_	0.487 ***	0.413	0.560	0.038	12.953	<0.001
S_(ADL)_ ↔ S_(depression)_	0.844 ***	0.574	1.113	0.138	6.135	<0.001
I_(ADL)_ → S_(depression)_	−0.282 ***	−0.406	−0.157	0.063	−4.440	<0.001
I_(depression)_ → S_(ADL)_	−0.053	−0.192	0.086	0.071	−0.743	0.457

Note: I_(ADL)_ = the intercept of activities of daily living; I_(depression)_ = the intercept of depression; S_(ADL)_ = the slope of activities of daily living; S_(depression)_ = the slope of depression; *** *p* < 0.001.

**Table 5:** Decomposition of effects and mediating effects of ADL.

Influencing Paths	Standardized Effect Size	Proportion	Percentage (%)
Chronic diseases → depression	0.130	0.61	61%
Chronic diseases → ADL → depression	0.172 × 0.481 = 0.083	0.39	39%
Total effect	0.130 + 0.083 = 0.213	1.00	100%

Note: ADL = activities of daily living.

## Data Availability

The datasets used and/or analyzed during the current study are available from the author on reasonable request.
